# Opioid Mechanism Involvement in the Synergism Produced by the Combination of Diclofenac and Caffeine in the Formalin Model

**DOI:** 10.1155/2013/196429

**Published:** 2013-05-09

**Authors:** José María Flores-Ramos, M. Irene Díaz-Reval

**Affiliations:** Centro Universitario de Investigaciones Biomédicas, Universidad de Colima, Avenida 25 de Julio No. 965, Colonia Villa San Sebastián, 28045 Colima, Col, Mexico

## Abstract

Analgesics can be administered in combination with caffeine for improved analgesic effectiveness in a process known as synergism. The mechanisms by which these combinations produce synergism are not yet fully understood. The aim of this study was to analyze whether the administration of diclofenac combined with caffeine produced antinociceptive synergism and whether opioid mechanisms played a role in this event. The formalin model was used to evaluate the antinociception produced by the oral administration of diclofenac, caffeine, or their combination. Opioid involvement was analyzed through intracerebroventricular (i.c.v.) administration of naloxone followed by the oral administration of the study drugs. Diclofenac presented a dose-dependent effect, with a mean effective dose (ED_50_) of 6.7 mg/kg. Caffeine presented an analgesic effect with a 17–36% range. The combination of subeffective doses of each of the two drugs presented the greatest synergism with an effect of 57.7 ± 5.6%. The maximal antinociceptive effect was obtained with the combination of 10.0 mg/kg diclofenac and 1.0 mg/kg of caffeine, with an effect of 76.7 ± 5.6%. The i.c.v. administration of naloxone inhibited the effect of diclofenac, both separately and combined. In conclusion, caffeine produces antinociceptive synergism when administered in combination with diclofenac, and this synergism is partially mediated by opioid mechanisms at the central level.

## 1. Introduction

Diclofenac (DIC) is a nonsteroidal anti-inflammatory analgesic that is widely used for treating arthritic-type diseases, for acute pain, and during the postoperative period [1]. In addition to its known action mechanism that inhibits prostaglandin synthesis [2] and that is shared with the other nonsteroidal analgesics, studies published demonstrate that diclofenac activates other action mechanisms that contribute to its analgesic effect. These mechanisms involve the participation of endogenous opioids [3, 4], serotonin [5], noradrenalin [6, 7], the L-arginine-nitric oxide-cGMP pathway [8–10], and the activation of potassium channels [11]. Diclofenac is more effective than acetylsalicylic acid; nevertheless, its administration for long periods of time is not recommendable, due to the adverse gastrointestinal effects that are characteristic of this drug group. 

The clinical use of drug combinations has been employed for different purposes, such as increasing therapeutic effectiveness and reducing adverse effect, both of which are extremely important in improving patient health. When a combination of drugs is administered, effectiveness is altered in different ways: the final effect may be equal to the sum of the effects of all the drugs administered separately (additive effect), it may be less than the sum of the effects of the drugs administered independently of each other (antagonistic effect), and optimally, the final effect may be greater than the sum of the effects of the separately administered drugs, which is known as synergism [12]. The combination of analgesics with different action mechanisms has been used for eliminating pain, as well as the combination of analgesics and adjuvant analgesics. Adjuvant analgesics are drugs that have no inherent analgesic effect and therefore are prescribed for other purposes. However, different experimental models with both rodents [13, 14] and humans [15] have demonstrated that when an adjuvant analgesic is combined with an analgesic, the effect of the analgesic is increased. 

Caffeine is one of the adjuvant analgesics that has been combined with certain nonsteroidal analgesics in order to increase their therapeutic effects [16]. This methylxanthine exerts various effects on the central and peripheral nervous systems through different mechanisms, such as intracellular calcium mobilization, phosphodiesterase inhibition, and adenosine-receptor blockade [17]. The aim of this study, using an inflammatory pain experimental model, was to analyze whether the administration of diclofenac combined with caffeine presented antinociceptive synergism, and whether opioid mechanisms were involved in its production. 

## 2. Materials and Methods

### 2.1. Animals

Male Wistar rats from our own laboratory animal center were used. They weighed between 180 and 200 g and were kept in a 12 h light-dark cycle environment. Food was removed twelve hours before the experiment, but they had free access to water. The animals were used only once and then euthanized. All experiments were carried out according to the norms of the Research and Ethics Committee of the International Association for the Study of Pain [18]; the guidelines and ethical standards for the study of Experimental Pain in Animals [19]; the guidelines of the Local Ethics Committee; and the technical specifications for the production, care, and use of laboratory animals of the Mexican Department of Agriculture, Livestock, and Rural Development NOM-062-ZOO-1999. 

### 2.2. Drugs

Diclofenac, caffeine, xylazine, naloxone, and formalin were obtained from Sigma-Aldrich, St. Louis, MO, USA. Ketamine was obtained from Pisa Laboratories (Mexico City, Mexico). All these drugs were dissolved in physiologic saline solution. 

### 2.3. Measurement of Antinociceptive Activity

The antinociceptive effect was evaluated using the formalin model [20], which consisted of administering 50 *μ*L of formalin at 2% in the subplantar region of one of the hind paws of the rat. After solution administration, the animals were placed in an observation chamber and nociceptive behavior was recorded. The evaluated behavior was number of flinches or paw shakes of the hind paw that had been injected with formalin. The flinches were recorded every five minutes for a total of one hour. This model is characterized by the presentation of a time course made up of two phases; the first phase lasts approximately 0 to 10 min, and then there is a rest period of about 5 min after which the second phase begins, ending after 60 min. This study only analyzed the antinociceptive effect in the second phase. 

### 2.4. Intracerebroventricular Injection

Action mechanism analysis was carried out by administering the opioid antagonist, naloxone (NX), by intracerebroventricular (i.c.v.) route, in accordance with the Paxinos and Watson method [21]. The rats were anesthetized with a mixture of ketamine and xylazine (75 and 12 mg/kg, resp.). They were placed in a stereotaxic apparatus and immediately afterwards a cannula was inserted into the right lateral ventricle; the lateral and anteroposterior coordinates were 1.8 mm and 0.8 mm, respectively. The rats were left in recovery for 48 h and on the day of the experiment they were given 12.13 *μ*g of i.c.v. NX, at a volume of 4 *μ*L.

### 2.5. Study Design

Different animal groups were given 0.316, 1.0, 3.16, 10.0, 31.6, and 56.2 mg/kg of diclofenac (DIC) and 1, 1.78, and 3.16 mg/kg of caffeine (CAF). Both drugs were administered orally, 30 min before receiving the formalin. In the following study phase, other animal groups were given the combination of DIC and CAF in all the indicated doses, resulting in a total of 18 combinations that were administered orally 30 min before the formalin. The control groups were given physiologic saline solution (the vehicle in which the drugs were administered) under the same conditions as the CAF, DIC, or their combination. 

Action mechanism analysis was carried out by administering 12.13 *μ*g of i.c.v. NX. Fifteen minutes later they received oral saline solution, CAF (1.78 mg/kg), DIC (31.6 mg/kg), or their combination (DIC 0.316 mg/kg + CAF 1 mg/kg). The control groups were given 4 *μ*L of i.c.v. saline solution, and then fifteen minutes later they received oral saline solution, CAF, DIC, or their combination at the same doses mentioned above. 

### 2.6. Data Presentation and Statistical Evaluation

The number of flinches per minute was the nociceptive response scored every 5 min from 15 to 60 min with regard to formalin administration to analyze the second phase. The time courses (TC) of nociceptive response of individual drugs and combinations were constructed by plotting the mean number of flinches as a function time. The cumulative nociceptive effect was analyzed and determined as area under curve (AUC) of the TC; AUC was obtained by trapezoidal rule [22]. Percentage of maximum possible effect (%MPE) was regarded as the antinociceptive effect and was calculated from the AUC obtained from the drug group (AUC_D_) and the control group (AUC_C_) with the following formula:

(1) $$\begin{array}{c} \%\text{MPE }=\;\left[\frac{\left({\text{AUC}}_{\text{D}}-{\text{AUC}}_{\text{C}}\right)}{{\text{AUC}}_{\text{C}}}\right]\times 100. \end{array}$$

On the basis of the addition of the effects of the individual component drugs, a %MPE equivalent to the sum was expected. If the %MPE of the combination was greater than the sum of the corresponding individual %MPEs, the result was considered to show synergism; if it was similar to the sum, it was considered to show an additive antinociceptive effect [23]. The %MPE values obtained from the antinociceptive effects produced by either diclofenac or caffeine (assayed separately) were compared with the %MPE value obtained from the corresponding combination by ANOVA, followed by a Tukey test. The synergistic effects between diclofenac and caffeine were evaluated by a two-way ANOVA. The values were expressed as the mean of six determinations ± S.E.M. The results were considered statistically significant when *P* < 0.05.

## 3. Results

**Figure 1:**
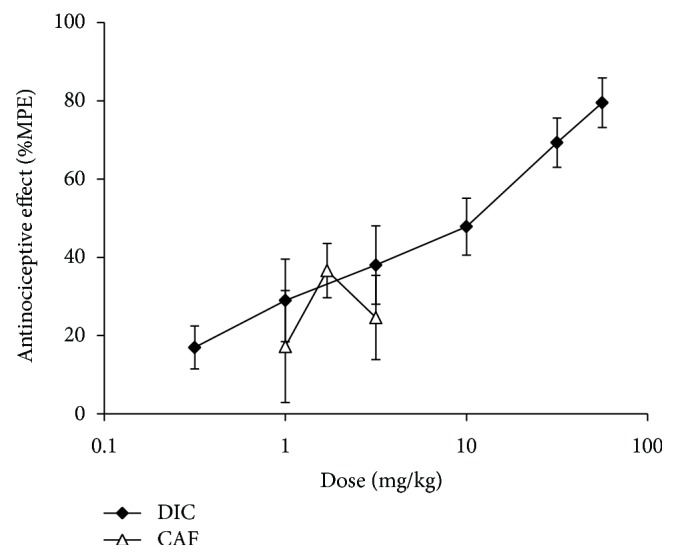
The DRC of oral diclofenac (DIC) in doses of 0.316, 1.0, 3.16, 10.0, 31.6, and 56.2 mg/kg and oral caffeine (CAF) in doses of 1, 1.78, and 3.16 mg/kg. For each dose, the mean of 6 determinations ± S.E.M. was plotted on the graph.

The antinociceptive effect of DIC was analyzed using the formalin model, in which the evaluated nociceptive behavior was flinching of the hind paw that had received the formalin. Nociceptive behavior decreased at increasing doses of DIC; in other words, the antinociceptive effect increased, presenting a dose-dependent effect at a dose ranging from 0.316 to 56.2 mg/kg, with a maximum effect of 79.56 ± 6.32%. The dose-response curve (DRC) is shown in Figure 1. Mean effective dose (ED_50_) for DIC under these experimental conditions was 6.7 mg/kg. In another animal group the antinociceptive effect of CAF was evaluated at doses of 1, 1.7, and 3.16 mg/kg. As can be observed in the graph, those doses did not present important antinociception and there were no statistically significant differences among those effects. Therefore, under these experimental conditions, caffeine did not present a dose-dependent antinociceptive effect. 

**Figure 2:**
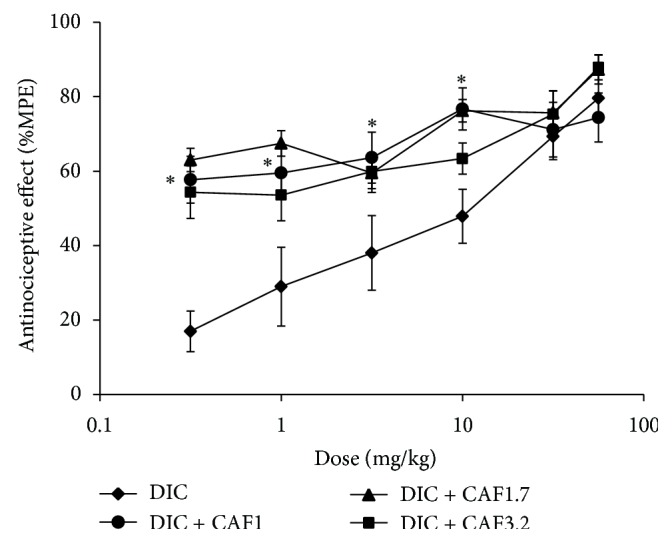
The DRC of the combinations of diclofenac (DIC) and caffeine (CAF) compared with the DRC of DIC administered separately. Both drugs were administered orally. Each dose represents the mean of an *n* = 6 ± S.E.M. ^*^ *P* < 0.05 group that was administered with combination (DIC + CAF 1 mg/kg) versus the animals that only received DIC.

The effect of caffeine on diclofenac-induced antinociception was analyzed by administering each of the CAF doses with the 6 DIC doses, resulting in three more DRCs. These are shown in Figure 2. This graph demonstrates that when DIC was combined with CAF, the effect increased, especially in the first doses. This increase was important because the doses of 0.316, 1, and 3.16 mg/kg of DIC produced minimal effects that were within the 16–38% effect range. And when DIC was coadministered with minimum doses of caffeine, that were also subeffective, the effect increased to a 54–76% range. All three combination DRCs shifted upwards. However, the most important curve was the one representing the combination with the 1 mg/kg caffeine dose, because it was statistically significant (*P* < 0.05) for the first four doses, meaning that these combinations presented synergism. The other two curves showed an additive effect in the first two doses, and the remaining combinations presented an antagonistic effect. 

**Figure 3:**
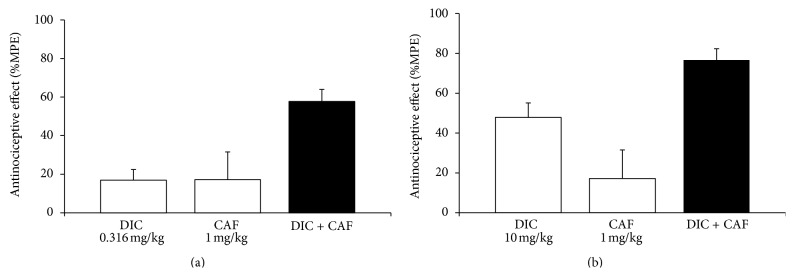
The effect of caffeine (CAF) administered in combination with diclofenac (DIC) in the formalin model. (a) The white bars show the effect of DIC (0.316 mg/kg) and CAF (1 mg/kg) when administered separately by oral route; the black bar shows the effect of the drug combinations at the indicated doses. (b) The white bars show the effect of DIC (10 mg/kg) and CAF (1 mg/kg) when administered separately by oral route; the black bar shows the effect of the drug combinations at the indicated doses. For each treatment the mean of an *n* = 6 ± S.E.M. was plotted on the graph.

In the synergism analysis it was seen that the combination that presented the greatest supraadditive effect was that of 0.316 mg/kg of DIC + 1 mg/kg of CAF, which was two times greater than the sum of the effects of the drugs administered separately.  Figure 3(a) shows the antinociceptive effects of the separate administration of the two drugs and there is practically no effect with either dose. However, an effect of 58 ± 6.3% was reached when they were administered in combined form. Maximum effectiveness (Figure 3(b)) was seen with another important combination; under those experimental conditions, the doses of the administered drugs were 10 mg/kg for DIC and 1 mg/kg for CAF, which exhibited an antinociceptive effect of 77 ± 5.6%. This was similar to the effect of the highest DIC dose administered separately that was used under these same experimental conditions. The percentage of antinociception was 79.5 ± 6.3% with a DIC dose of 56.6 mg/kg; this dose was five times higher than the DIC dose used in the drug combination. 

**Figure 4:**
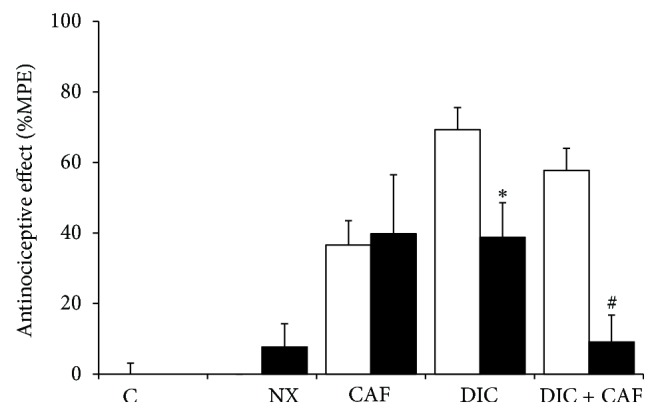
The effect of i.c.v. naloxone (NX, 12.13 µg/rat) on the antinociception produced by diclofenac (DIC, 31.6mg/kg), caffeine (CAF, 1.78 mg/kg), or their combination (DIC 0.316 mg/kg and CAF 1 mg/kg) is shown by the black bars compared with the same drugs with i.c.v. saline solution (white bars). The mean of six determinations ± S.E.M. was plotted on the graph. ^*^ *P* < 0.05 for the group with DIC but without NX. ^#^ *P* < 0.05 for the group with DIC + CAF but without NX.

For the purpose of analyzing whether synergism was a product of pharmacodynamic mechanisms, the opioid antagonist, naloxone (NX), was administered at a dose of 12.13 *μ*g/per rat, i.c.v., at a volume of 4 *μ*L, before the separate or combined administration of the two drugs.  Figure 4 shows that NX did not present an antinociceptive effect; other animal groups were given the same pretreatment with the antagonist, after which 1.78 mg/kg of CAF or 31.6 mg/kg of DIC was administered. The effect was not altered in the caffeine group, while a statistically significant 44% decrease in effect was observed in the diclofenac group. These data suggest that when caffeine is administered separately it does not activate opioid mechanisms, but in contrast, diclofenac does. 

Similarly, when the antagonist was administered to the group that was treated with the combination that presented the greatest synergism, the antinociceptive effect was significantly inhibited and the antinociception inhibition percentage produced by NX was 84.3%, a significantly higher percentage than that observed when diclofenac was administered separately. The combination presented an effect of 57.7 ± 6.3% and an antinociception percentage of 9.1 ± 7.6% with the antagonist was observed. These data suggest that the combination acts through opioid mechanisms to produce synergism. 

## 4. Discussion 

The formalin model is an experimental model that induces antinociception through a chemical stimulus (2% formalin) in the hind paw of the rat. Nociception is characterized by the shaking of the paw immediately after solution administration due to the direct nociceptor stimulation by formalin. Approximately 10–15 min after administration there is a rest period, after which nociceptive behavior begins again and lasts up to 60 min. This second nociception phase has been reported to take place due to the presence of inflammatory mediators that sensitize the nociceptors. It is a well-established characteristic of this model that nonsteroidal anti-inflammatory drugs (NSAIDs) present an antinociceptive effect in this second phase [24]. For this reason, the present study only reported the results of that phase. 

The nonsteroidal anti-inflammatory analgesic, diclofenac, presented a dose-dependent effect in the second phase of the formalin model, which is a behavior that has already been reported in other studies using this model [25]. On the other hand, caffeine is a stimulant of the central nervous system and it has been proven in some experimental pain models, including the formalin model, that it does present an antinociceptive effect [26], while in humans, it has only been shown to be effective in relation to headache [27]. Under the experimental conditions of the present study, caffeine was administered in minimum doses that did not present an antinociceptive effect, even when only a 2% formalin concentration was used; it has already been reported that the ED_50_ for caffeine is 5 mg/kg, when 2% formalin is administered to induce nociception [28]. Under our experimental conditions, caffeine doses were lower than 5 mg/kg and the effects were lower than 50%, all of which concur with the published reports. 

When the combinations of both drugs were administered, it was clearly seen that caffeine was capable of increasing the effect of diclofenac, especially in the first doses, in such a way that the minimum doses of the two drugs combined had an increased effect, when in their separate administrations they had no effect, resulting in an ED_50_ of 6.715 mg/kg for diclofenac. The minimum effect of the drug combination was above 50% with 0.316 mg/kg of diclofenac combined with any of the three caffeine doses used in the study. Likewise, the maximum antinociceptive effect was 76.7 ± 5.7% with 10 mg/kg of DIC combined with 1 mg/kg of caffeine. In both cases, the diclofenac dose was small, making it possible that adverse effects in chronic treatments may not be as severe as those that currently exist at the commonly prescribed doses. 

Other studies have been carried out on NSAID and caffeine combinations, in which no antinociceptive effect on the part of separately administered caffeine was observed, due to the fact that the pain stimulus was different because nociception was induced through uric acid administration (the PIFIR model). These studies had similar results to ours, in the sense that when various analgesic doses were combined with increasing doses of caffeine, DRCs were formed that shifted upwards and to the left so that the ED_50_ of the analgesic decreased. This indicated the potentiation of, and increase in, the effect of the drug combination [14]. In the studies carried out with the PIFIR model, the synergism analysis showed that not all studied NSAID combinations with caffeine presented potentiation of the effect. Some only presented an additive effect, and others even presented antagonism. We observed the same results in our study, in which synergism was achieved in only four of the combinations, four other combinations presented an additive effect, and the remaining combinations presented an antagonistic effect. This is worth emphasizing because it used to be thought that caffeine did not increase the effect of analgesics [16]. That it does has been confirmed for certain NSAIDs, but not at all doses, and therefore it is important to determine the proportions that produce synergism. 

Adverse effects were not analyzed in this study. However, in other studies, adverse effects at the gastrointestinal level have been reported [29]. And the combination doses that produce a supraadditive synergism of the antinociceptive effect have been observed to generally be doses lower than those that produce maximum effects when administered separately, and ulcers and gastric erosions tend to be fewer with the drug combinations. 

Diclofenac is a nonsteroidal anti-inflammatory analgesic whose principal action mechanism, like all the other analgesics of this group, is the inhibition of prostaglandin synthesis. But it was also reported that it produced an antinociceptive effect in the abdominal writhing model when directly administered into diverse nuclei of the brain. This suggests that it activates other mechanisms different from prostaglandin synthesis inhibition. Other experiments have shown that with NX and DIC i.c.v. administration, the antinociceptive effect was inhibited and therefore those authors concluded that diclofenac is capable of interacting with opioids at the central level [30]. The results of the present work concur with those reports, because when oral diclofenac was administered to animals that had received pretreatment with i.c.v. NX, antinociception decreased by 44%, suggesting an important opioid mechanism participation. But it also indicates that other mechanisms were being activated. Previous reports have stated that serotoninergic antagonists also inhibit the effect of diclofenac [30]. Other researchers have reported that this analgesic activates the L-arginine-nitric oxide-cGMP pathway [10]. 

Caffeine also activates different mechanisms, but due to the plasmatic concentrations that are reached through its normal dietary consumption, the most probable mechanism is adenosine-receptor antagonism. In regard to opioids, there are studies that show that caffeine does not interact with them to produce an antinociceptive effect in the experimental models in which antinociception with caffeine has been observed [31]. The i.c.v. administration of NX did not alter the antinociceptive effect in our inflammatory pain model and so the present results concur with those previously reported. 

The above mentioned studies provide evidence that diclofenac acts on both the peripheral and the central levels, and when it is combined with caffeine there is significant synergism with certain combinations. In an attempt to understand the participating pharmacodynamic mechanisms, NX was administered i.c.v., followed by the combination, and an 84% decrease was observed. This was greater in relation to the inhibition seen when diclofenac was administered separately. These data suggest that the combined drugs, through different mechanisms, end up leading to greater opioid participation than when the analgesic is administered separately. It is possible that their mechanisms interact at some point in signaling cascades that are finally observed in the entire animal as an important increase in effect, that is to say, as supraadditive synergism. 

In addition, the pharmacokinetics of the two drugs should not be ignored, because given the fact that studies on diclofenac-caffeine interaction have not been carried out, it is possible that the synergism that has been observed was being produced by pharmacokinetic mechanisms such as an increase in absorption or some alteration in metabolism or elimination that as a result produced an increase in the plasmatic concentrations of diclofenac. Nevertheless, there are studies on NSAID combinations with caffeine in which analgesic plasmatic concentrations were not altered [32]. Therefore, with the results of the present study, we conclude that caffeine produces synergism with doses of diclofenac that are subeffective when the drug is administered separately, resulting in a possible benefit for patients that need chronic diclofenac administration. Our results also showed that this synergism is produced, at least in part, through opioid mechanisms.
